# Leishmaniases diagnosis: an update on the use of immunological and molecular tools

**DOI:** 10.1186/s13578-015-0021-2

**Published:** 2015-06-17

**Authors:** Milena de Paiva-Cavalcanti, Rayana Carla Silva de Morais, Rômulo Pessoa-e-Silva, Lays Adrianne Mendonça Trajano-Silva, Suênia da Cunha Gonçalves-de-Albuquerque, Diego de Hollanda Cavalcanti Tavares, Maria Carolina Accioly Brelaz-de-Castro, Rafael de Freitas e Silva, Valéria Rêgo Alves Pereira

**Affiliations:** Department of Immunology, Aggeu Magalhães Research Center, Oswaldo Cruz Foundation, Av. Prof. Moraes Rego s / n, 50670-420 Recife, PE Brazil; Department of Natural and Exact Sciences, University of Pernambuco (UPE), St. Capitão Pedro Rodrigues, 105, 55920-000 São José, Garanhuns, PE Brazil

**Keywords:** Visceral leishmaniasis, Cutaneous leishmaniasis, Diagnosis, Immunological tools, Molecular tools

## Abstract

Leishmaniases are caused by obligate intracellular protozoan parasites of the genus *Leishmania*. They cause a spectrum of diseases, most notably visceral (VL), cutaneous (CL), and mucosal (ML) leishmaniasis, which affect millions of people around the world, each year. Despite scientific advances, leishmaniases cases are expanding, constituting an important public health problem. Immunological and molecular diagnostic tools have been increasingly applied for the early detection of these parasitic infections, since the existence of limitations in clinical and parasitological examinations may provide false results, thus interfering in epidemiological research and diseases control. Although there is a great diversity of available immunological assays, important common deficiencies persist, which explains the current exploration of the molecular biology in research fields, especially the Polymerase Chain Reaction (PCR) and its variants, such as real-time quantitative PCR. However, in the last years, significant results have also been reached inside of immunological context (especially by Flow Cytometry), for humans and dogs, demonstrated by research works of the New and Old worlds. In spite of their potential to clarify and minimize the present global situation of the diseases, the implementation of molecular or immunological innovative reference assays for VL and CL at health services is still a challenge due to several reasons, including lack of standardization among laboratories and structural concerns. In this article we bring classical and current information about technological advances for the immunological and molecular leishmaniases diagnosis, their features, and applications.

## Introduction

Leishmaniases are caused by obligate intracellular protozoan parasites of the genus *Leishmania*, capable of developing a spectrum of diseases, most notably visceral (VL), cutaneous (CL), and mucosal (ML) leishmaniasis [1, 2]. Global VL and CL incidences are approximately 0.2–0.4 million and 0.7–1.2 million cases occurring each year, respectively [3]. Despite the increasing number of infected individuals being registered in several countries of the Americas (New world) Europe, Asia and Africa (Old world), a huge number of cases are believed to be under-reported [4].

Leishmaniases are still ignored in discussions of tropical disease priorities, even being estimated to cause the ninth largest disease burden among individual infectious diseases [5, 6]. Despite scientific advances, leishmaniases constitute an important public health problem. Up to the moment, the leishmaniases diagnosis is performed by an association of clinical, epidemiological and laboratorial data. Particularly in relation to laboratorial methods, the lack of a gold-standard for human patients or animals is a limitation for the disease control, because the achievement of accurate epidemiological data is associated with the guidance of control measures, thus helping to increase their efficiency. Furthermore, false-negative results could delay early implementation of treatment, thereby contributing for maintenance of reservoirs, and as a consequence, the preservation of parasitological cycles in their environment.

Rapid methods for diagnosis and species identification are urgently needed, along with therapies, prophylactics, and control measures that are effective, safe, affordable, and easily administered [2]. In this context, this article provides an overview on classical diagnosis and current information about technological advances for the immunological and molecular leishmaniases diagnosis, their features, applications, limitations and perspectives.

## Immunological tools for visceral and cutaneous leishmaniasis diagnosis

The immunological procedures have been employed for screening and for the definitive diagnosis of diverse parasitological illnesses because of their easiness and accuracy. For leishmaniases, these methods are largely applied, being, in some routine protocols, the single diagnostic principle, before anti-*Leishmania* drug prescription [7]. Nonetheless, important limitations may carry to incorrect diagnostic interpretation, like cross-reactions, especially with phylogenetically closely related species, and loss of accuracy in immunosuppressed patients. The combination of different techniques, like immunochromatographic test (ICT) and rapid immunoenzyme assay, or the employment of different antigenic targets and the combination of distinct recombinant proteins in the same assay are strategies that may improve the global specificity and sensitivity, thus bringing a higher accuracy to the routine protocols [8–10]. The association with the molecular biology, mainly in reference centers, has become a common approach to the differential diagnosis of leishmaniases, especially when parasitology is also inconclusive [11, 12]. Even so, researchers around the world have been developing new protocols and technologies to the continuous enhancement of the immunological diagnosis, thus ensuring smaller risks to patients in the detection of leishmaniases.

### Classical immunological tools

Montenegro skin test (MST) has been successfully used in the diagnosis of cutaneous forms, but is negative for recent lesions, in the diffuse form, and also in immunosuppressed patients [13]. The test is commonly positive in endemic areas due to the occurrence of subclinical infections. Furthermore, other characteristics may hamper the applicability of the method: cross-reactions; long time to retest, if necessary (at least two years); false-positive results caused by lack of patient cooperation, which itches the application site; subjectivity of the reading (especially when the diameter of the induration has between 4 and 5 mm) and so on [14]. In the diagnosis of CL the use of indirect immunofluorescence assay (IFA) associated with MST or a parasitological technique is recommended to provide a differential diagnosis. The limitation of IFA lies in the fact that it does not correlate the levels of circulating antibodies with disease staging. In addition, there is the possibility of cross reactivity with other trypanosomatids and fungi [15–17]. IFA is based on the detection of anti-*leishmania* antibodies by employing specific antigens (promastigote form, normally) and secondary antibodies (anti-immunoglobulin antibody) conjugated with a fluorescent dye [16, 17]. The technique is becoming less explored in routine not only for CL diagnosis, but also for canine VL (CVL) diagnosis mainly because of its low specificity, in contrast with the high sensitivity. Incompatibility or poor reaction between the secondary and primary antibodies or the antigen are also constraints associated with this indirect immunological assay.

The majority of the immunological techniques for detection of anti-*Leishmania* antibodies has been based on reactions like Enzyme-Linked Immunosorbent Assay (ELISA) [11, 18]. The sensitivity and specificity of ELISA depends on the antigen used [11]. In this context, several antigens with different molecular weights have been identified for potential use in the diagnosis. Recombinant *Leishmania* protein K39 (rK39), a very important and broadly employed antigen, showed 100 % specificity and 96 % sensitivity for the diagnosis of VL [19]. An interesting feature of this antigen is that it can be used in patients co-infected with HIV, in which anti-K39 antibody levels decline rapidly with the treatment success [11, 20, 21]. Other candidates for the diagnosis of several forms of leishmaniases are recombinant or purified membrane glycoproteins (gp): gp63, gp70, and gp72, and A2 protein, all of which are specific for the genus *Leishmania*. A2 protein is present in amastigotes, and studies suggest that is particularly useful for diagnosis of canine VL [20, 21]. The use of recombinant or purified gp63, gp72 or gp70 improved the sensitivity and specificity of ELISA. However, cross-reactions with other diseases caused by trypanosomes may occur [22, 23].

The numerous commercial kits of rapid diagnostic tests (RDTs) available are largely explored nowadays in routine. The easiness concerning its use and interpretation, as well as the quick results have contributed to its broad application, especially for screening. The high stability in a broad range of temperature (generally 4–30 °C) facilitates its employment in field for screening of CVL. RDTs are based on the detection of specific antibodies in serum or peripheral blood of the patient with VL. An important RDT, known as TRALd (Rapid Antibody Test *Leishmania donovani*) was developed using two recombinant proteins, the rK39 and K26, fixed on nitrocellulose paper. An initial clinical evaluation of TRALd observed 100 % of sensitivity and 98 % of specificity [11, 21, 23, 24]. Even when antigenic combinations are used, there is the possibility of cross-reactions with other trypanosomatids, being then indicated the application of a confirmatory exam, normally of a distinct principle (e.g. IFA, ELISA etc.) or distinct antigenic composition.

### New immunological tools

In relation to the new immunological tools, the use of flow cytometry (FC) in the diagnosis of canine VL through the detection of anti-fixed *L. infantum* antibodies has been increasingly explored [25]*.* Researchers demonstrated an excellent performance of an FC prototype in canine VL diagnostic, with high specificity, sensitivity, predictive values and accuracy, even when animals were infected with other pathogens (such as *Trypanosoma cruzi* and *Babesia canis*), and also absence of false-positive results in vaccinated dogs [26]. Recently it was demonstrated the potential of a magnetic microsphere coated with *Leishmania* recombinant antigens associated with FC as a viable tool for a highly sensitive laboratorial serodiagnosis of both clinical and subclinical forms of canine disease [27]. For human form, it was shown the detection of specific IgG antibodies against *L. chagasi* using FC for cure assessment [28].

FC to detect anti-live *L. braziliensis* antibodies has been first described by Rocha *et al.* [29], in which they demonstrated 93.6 % sensitivity in patients with active disease. Researchers working with live and fixed *L. braziliensis* showed that FC can be a useful serological technique to detect anti- *L. braziliensis* IgG antibodies, with the antigens displaying an 86 and 90 % sensitivity, respectively [30]. A good performance using fixed *L. amazonensis* promastigotes was also demonstrated [31]. Oliveira *et al.* [32] showed that FC had a better performance compared to IFA in the monitoring of specific post therapeutic cure of CL. Therefore, FC is becoming an increasingly useful tool in health care and research laboratories, due to its accuracy and reproducibility. Although there is still a substantial cost regarding the operational support in experiments involving FC, Shared Resource Laboratory models are enhancing the scope and quality of scientific research that applies the FC based methodologies [26, 33, 34].

The Table 1 summarizes the main aspects addressed in the immunological methods used in leishmaniases diagnosis.

**Table 1:** Advantages and limitations of immunological methods used in leishmaniases diagnosis

Method	Antigen	Advantage	Limitation
Montenegro skin test	Killed whole parasites	Low cost and detection of T cell immunity	May not detect cases of visceral leishmaniasis in some stages of the disease. Cannot differentiate between infection and disease, nor active and progressive disease. Risk of recurrence.
Enzyme-Linked Immunoabsorbent Assay (ELISA)	Recombinant molecules	Low cost and high sensitivity and specificity	Sensitivity and specificity is highly dependent on the antigen used
Immunofluorescence	Killed whole parasites	High sensitivity and specificity	Laborious process, time and cost consuming. Need of trained personnel to perform the test.
Flow cytometry	Recombinant molecules and/or killed whole parasites have been tested	Better sensitivity and specificity when compared with all other methods. Small amount of blood. Can differentiate between infection and disease, and cured patients.	Cost associated with reagents and equipment. Few studies yet.
Rapid Antibody Test (RAT)	Recombinant molecules	Low cost, small amount of blood, fast	Sensitivity and specificity is highly dependent on the antigen used
Direct Agglutination Test (DAT)	Killed whole parasites	Low cost, small amount of blood	Need of long incubation time, well-trained laboratory technicians, antigen cost, and quality controlled antigen

## Molecular tools for leishmaniases diagnosis

The limitations demonstrated by the conventional techniques, both parasitological and serological, have led the scientists to an increasing exploration of the molecular biology as a complement, as well as an alternative for the accurate diagnosis of leishmaniases. The practicality, safety and reliability of the molecular tools, in addition to the wide number of applications and the promising results have contributed to the continuous acceptance of these methods in routine and reference laboratories around the world.

The Polymerase Chain Reaction (PCR) technique and its variations, like Nested-PCR (nPCR), Seminested-PCR (snPCR) and Quantitative Real Time PCR (qPCR) have been largely employed for the optimization of new diagnostic assays, using different target regions and samples [35–37]. The follow-up of the treatment aiming the evaluation of a drug efficacy is a common approach [38] proportionated by the qPCR, with its capacity to estimate the parasitological burden in several specimen types [39, 40]. Species characterization of *Leishmania* is also an important application of the PCR, and it has been strongly explored nowadays [41, 42]. Coupled with different methodologies, including gene sequencing and Restriction Fragment Length Polymorphism (RFLP) analysis, the studies have brought this kind of analysis with distinct goals: species confirmation in epidemiological researches [43, 44], specificity assessment of new optimized assays [11, 45] and comparative studies [46], for example. Ozensoy-Toz *et al.* [47] have used fluorescent dyes and the resulting melting temperature (Tm) interpretation as criterion for *Leishmania* species differentiation, to both CL and VL etiological agents, with success. The author, as others [43, 48], also performed phylogenetic analysis of species from isolates by gene amplification and sequencing. According to Grimaldi and Tesh [49], the correct identification of the causative *Leishmania* species is directly related to decision upon the appropriate treatment regimens and to design effective control programs.

### Visceral leishmaniasis diagnosis

Between 2011 and 2013, conventional PCR protocols for VL presented a sensitivity variation from 53.7 to 97.78 % for humans and from 72.2 to 98.7 % for dogs, and the specificity varied from 61.82 to 100 % for humans and from 83.3 to 96.4 % for dogs [41, 43, 50, 51]. The high variability also occurs to qPCR for humans, with a specificity variation from 29.6 to 100 %. The range of sensitivity (91.3–100 %) [41, 51] presented short and with high values, thus demonstrating the applicability of the qPCR at situations in which a sensitive tool is pivotal, like disease monitoring to predict relapse. Results obtained by Solcà *et al.* [52] show the PCR as being more sensitive than qPCR in dog samples. This reinforces that some characteristics like the DNA target region, as well as the pair of primers used may be determinant.

As occurs in different clinical statuses, the parasitic burden in the various samples differs. Getting advantage on the specimen type, Pandey *et al.* [53] and Silva *et al.* [54] standardized PCR reactions (snPCR and qPCR, respectively) to detect the parasite DNA from Giemsa-stained smears, prepared from bone marrow aspirates. The first author’s assay detected *L. donovani* DNA in 68.7 % of the human samples. The second author detected *L. chagasi* DNA in 100 % of the canine samples. Both assays presented superiority in relation to microscopy and culture. Other alternative samples, like urine and conjunctival swabs are being largely explored for humans [55, 56] and dogs [39, 57–60] with good accuracy. Although the small parasite DNA amount, they do not request invasive procedures for obtaining, thus bringing security and comfort to the patient [36, 57].

Despite these advantages, several *Taq* Polymerase inhibitors are found in these clinical specimens or they are commonly used for sample collection and DNA extraction, like EDTA, Proteinase K, Phenol and high salts concentrations [61, 62]. Recently, some authors have brought the strategy of the multiplex PCR to include endogenous controls altogether with the detection system of *Leishmania* sp., in the same reaction, for PCR [61] and qPCR [63] assays. Mohammadiha *et al.* [41] performed this multiplex format for humans and dogs, using TaqMan-based qPCR, having reached a very good sensitivity in both cases (93.9–100 %, respectively). Gonçalves-de-Albuquerque *et al.* [64] standardized a triplex PCR to CL capable to monitor not only the sample quality, but also small losses of DNA during the extraction process by using a plasmid as reporter.

Innovative molecular approaches, as the Nucleic Acid Sequence-Based Assay (NASBA), the Loop-Mediated Isothermal Amplification (LAMP), and the low-tech Oligo-TesT have been increasingly applied for *Leishmania* DNA or RNA detection. The NASBA have its variations, the quantitative (QT-NASBA) and coupled to oligochromatography (NASBA-OC) [65–69]. Vries *et al.* [65] have used QT-NASBA for the evaluation of the efficacy of a drug for VL treatment, by estimating the quantification of *L. infantum* parasites in blood. Basiye *et al.* [66] and Mugasa *et al.* [67] have applied NASBA-OC to diagnostic assays development, based on *L. donovani* RNA detection, reaching sensitivity of 79.80–93.30 % (respectively) and specificity of 100 % (both). The NASBA-OC uses a sensitized membrane of an oligochromatographic dipstick to detect the amplified RNA, in 5–10 min just with a pipette and a water bath, but without quantification capacity [68]. QT-NASBA has the inconvenience of the electrochemiluminescence as tool of detection, which involves more handling steps and procedure time than qPCR and Reverse transcription-qPCR (RT-qPCR) [69].

The LAMP, a promising diagnostic tool, has been adopted as an alternative technique to PCR, since it is a faster, sensitive and less expensive technology, which uses the turbidity of the sample as criteria of positivity. There is no need of a thermal cycler, just a water bath or a heat block, since the reaction is isothermal. Therefore, this is a tool suitable for field application [70, 71]. Verma *et al.* [72] developed a LAMP-based assay for *L. donovani* detection in humans with VL and Post-Kala-azar Dermal Leishmaniasis (PKDL) in which the sensitivity and specificity rates were good to both cases, achieving 96.4–98.5 % (in VL blood samples); 96.8–98.5 % (in tissue biopsy samples). Nevertheless, Chaouch *et al.* [73] developed a LAMP amplification for *L. infantum* detection in dogs, and the sensitivity reached was low (54.2 %), though having performed better than IF and PCR, statistically. The chosen target (cysteine Protease B gene - *cpb*) and the non-use of an internal quality control can in part explain this result.

### Cutaneous leishmaniasis diagnosis

As presented for VL, molecular techniques are advancing in studies for increasing their sensibility and specificity. They have been increasingly recommended for CL diagnosis due to their accuracy and speed [74], when compared to conventional diagnostic methods [47, 74]. Mohaghegh *et al.* [75] used PCR for confirming negative direct stained smear, confirming the higher sensitivity of the molecular method in comparison with classical diagnosis (direct stained smear). As for VL, different biological samples can be used with the molecular technology: blood [76], smear [75], scarification of the edge lesion [77] and biopsy skin [78, 79].

Several authors have used the qPCR technology to detect the DNA from the etiological agents in varied samples from animals and human patients, allowing studies related to parasite load, host-parasite interaction, monitoring of therapy and post-treatment response in infected human patients [80, 81]. Another common approach to CL is the application for differentiation of *Leishmania* species [47, 81–83]. Authors using dyes or fluorescent probes have had great results. Paiva-Cavalcanti *et al.* [82] used skin samples from humans and blood samples from domestic animals; Ozensoy Toz *et al.* [47] used skin aspiration fluid, smear, and biopsy from human patients; Pita-Pereira *et al.* [83] used skin biopsy samples from patients living in areas with well-known occurrences of CL. They have differentiated *Viannia* and *Leishmania* subgenera through Tm. Nonetheless, the qPCR requires a laboratory with the technical capacity to perform it; thus, this technology is becoming available at Central Diagnostic Laboratories, in countries where leishmaniases are endemic [81].

The nPCR is commonly used for some researchers [78]. Shirian *et al.* [84] performed the technique by using scraped off of slides with impression smears from 20 suspect cases of ML, getting 18 positive samples (90 %). Only eight were positive through direct microscopy (44.4 %). The PCR based method was not only a useful and more precise diagnostic approach in the identification of ML cases with negative cytology, but also showed to be efficient in determining the species of the parasites. Azzi *et al.* [74] concluded that nPCR is a useful technique for studying the molecular epidemiology in the field.

The QT-NASBA assay is a useful instrument to monitor parasite load in skin biopsies of patients with CL after treatment and can help to predict clinical outcome [69]. qPCR, RT-qPCR and QT-NASBA were compared by Van der Meide *et al.* [69], and they concluded that RT-qPCR and QT-NASBA are the most sensitive assays, generating reproducible results. However, as described before, QT-NASBA is less convenient since the electrochemiluminescence detection involves more handling steps and procedure time.

Despite the variety of molecular tools (Pulsed Field Gel Electrophoresis – PFGE, the Multilocus Enzyme Electrophoresis – MEE, and more), PCR still represents one of the major advances on diagnosis and research of the leishmaniases [85], even presenting its deficiencies, which have been minimized due to the continuous effort of researchers, attempting to bring more viability to its application in health service.

Table 2 summarizes the main aspects addressed in the molecular methods used in leishmaniases diagnosis.

**Table 2:** Advantages and limitations of molecular methods used in leishmaniases diagnosis

Method	Advantage	Limitation
Conventional PCR (cPCR)	High sensitivity, specificity and accurate results. Many applications in molecular analysis. Easy diagnostic interpretation.	Unable to quantify the target DNA. Qualitative test. Time consuming. Limited detection range of some assays.
Quantitative real-time PCR (qPCR)	Higher sensitivity, specificity and security, quantitative capacity and speedy results. Possibility of species differentiation by melting temperature.	High cost due to equipment (thermocycler). Difficulty in interpreting the results, needing thus of a well-trained operator.
Nested-PCR (nPCR)	Higher specificity and sensitivity. Useful technique for studying the molecular epidemiology in the field.	Time consuming and higher cost. Unable to quantify the target DNA. Qualitative test.
Quantitative Nucleic Acid Sequence-Based Assay (QT-NASBA)	High specificity. It is based on an isothermal reaction and thus overcomes the need for a thermocycler; Ideal for lower-tech laboratories. Quantitative capacity. Indicated to detect active diseases; RNA detection.	It uses electrochemiluminescence as tool of detection, which involves more handling steps and procedure time. Assays developed only for RNA detection. . Few studies yet.
NASBA coupled with oligochromatography (NASBA-OC)	High specificity. Speedy results. There is no need of complex laboratorial structure. Simple dipstick format for the detection of amplification products. RNA detection.	Unable to quantify the target RNA. Assays developed only for RNA detection. Few studies yet.
Loop-Mediated Isothermal Amplification (LAMP)	High sensitivity. Low cost. Isothermal reaction, there is no need for a thermocycler. The temperature stability of the reagents enables its use in field conditions.	Unable to quantify the target DNA. Qualitative test. Few studies yet.

Table 3 gathers the clinical sensitivities and specificities of the immunological and molecular methods commented throughout the text.

**Table 3:** Clinical sensitivity and specificity of different immunological and molecular methods for diagnosis of leishmaniases

Method/clinical form	Specimen	Antigen/target	Sensitivity (%)	Specificity (%)	Reference (s)
Immunological tests:					
ELISA/VL	Human serum	rK39	96	100	[19]
TRALd/VL	Human serum	rK39, K26	100	98	[24]
FC-ALPA/CL	Human serum	Live *L. braziliensis* promastigotes	85.7–97.9	76.0–93.7	[29]
FC-ALPA-IgG/CL	Human serum	Live *L. braziliensis* promastigotes	86	78	[30]
FC-AFPA-IgG/CL	Human serum	Fixed *L. braziliensis* promastigotes	90	78	[30]
Molecular tests:					
cPCR/VL	Human blood	ITS-1, kDNA minicircle	53.7–97.78	61.82–100	[41, 51]
cPCR/VL	Canine blood	ITS-1, kDNA minicircle	72.2–98.7	83.3–96.4	[41, 43, 50]
qPCR/VL	Human blood	ITS-1, kDNA minicircle	91.3–100	29.6–100	[41, 51]
NASBA-OC/VL	Human blood	18S RNA; 18S DNA	79.8–93.3	100	[66, 67]
LAMP/VL	Human blood	kDNA minicircle	96.4	98.5	[72]
LAMP/PKDL	Human tissue biopsy	kDNA minicircle	96.8	98.5	[72]
LAMP/VL	Canine blood	cysteine Protease B (cpb)	38.2–69.5	65.2–89.5	[73]

*ELISA* Enzyme-linked immunosorbent assay, *TRALd* Rapid Antibody Test *Leishmania donovani. FC-ALPA/AFPA* flow cytometry anti-live/fixed promastigote antibody, *cPCR* conventional PCR, *qPCR* real-time quantitative PCR, *NASBA-OC* Nucleic Acid Sequence-Based Assay- oligochromatography, *LAMP* Loop-mediated isothermal amplification

### Molecular targets

Several different genomic targets are used for *Leishmania* sp. detection as rDNA (ITS-1 and SSU rDNA), kinetoplastid minicircle DNA (kDNA), splice leader mini-exon (SMLE), tryparedoxin peroxidase gene and, heat-shock protein 70 gene (HSP70) [47, 77, 79, 82, 86–89]. This is one of the challenges to be surpassed for a future implementation of a gold-standard molecular methodology for CL and VL. Nevertheless, the choice of each target is related to the different applicability, i.e., with the objectives of each study, such as the case of HSP70 used by authors for species typing [45, 89, 90], while the kDNA is the main target used for diagnostic screening [79, 91–93].

The kDNA is largely elected for DNA amplification of *L. donovani* complex, because of the high number of copies (10,000) per parasite [94]. This target has been chosen for several applications, like for epidemiological studies in canine populations, took place in countries like Brazil and China [43, 95, 96], for species characterization [43, 97], for treatment follow-up and for assays development, with great results [72, 98].

Rocha *et al.* [99] compared different PCR protocols for *Leishmania* subgenus species detection, based on kDNA minicircle or mini-exon amplification. Concerning the kDNA minicircle, as long as the PCR protocol for *L. amazonensis* pointed out no specificity, since *L. infantum* was also detected, the PCR assay for *L. infantum* did not amplify DNA of any *Viannia* spp. or *L. amazonensis*, showing thus the importance also in selecting a specific and representative region of the target DNA, for primers design or selection from other authors. The mini-exon protocol presented inability to recognize any *Leishmania* subgenus species. Intraspecific variations of the utilized strains were considered to explain it. Roelfsema *et al.* [42] have compared the mini-exon and the Internal Transcribed Spacer-1 (ITS-1) rDNA in molecular typing of clinical samples. The mini-exon has performed better for typing species belonging to *L. Viannia* subgenus by sequencing, whereas ITS-1 PCR has differentiated *L. infantum* and *L. donovani* after a Hae III RFLP analysis.

As kDNA, the ribosomal RNA small subunit gene (SSU rRNA), as well as the ITS-1 region have been explored for many objectives, including epidemiological researches in human and canine populations [40, 97, 100], assays optimization [35, 47, 70] and for studies involving HIV/VL co-infected patients, a situation in which the serological diagnosis of VL is limited, and the molecular diagnosis is highlighted [101, 102]. A qPCR system for CL created by Paiva-Cavalcanti *et al.* [82] using kDNA as target has applicability in population studies, etiological diagnosis and the monitoring of treatment efficacy of individual patients. The same work demonstrated the high concordance between kDNA and SSU rDNA, concluding that both may be used for American CL diagnosis; altogether, the results indicate the best performance of the kDNA protocol. Summarizing, the kDNA target may be used for diagnosis due to its sensitivity [78, 79, 87, 103–105], whereas ITS-1 is more specific and may be used for species identification [78].

Regarding the advances in molecular biology, the choice of the target region to be imaged is the key point for the successful use of diagnostic PCR-based technologies.

## Proposal of gold-standard diagnostic strategies for leishmnaniases detection in humans

Technological advances have contributed to raise new diagnostic guidelines not only in the parasitological field, but also in all areas regarding pathology. The technology has brought new analytical possibilities, with increasingly speed, accuracy and reliability. In microbiology, robotic-based equipments are helping to provide molecular assessments with safety and precision in both diagnostic routine and research. Regarding the leishmaniases, the advent of real-time PCR thermocyclers and flow-cytometric analysis (mainly) has contributed to the development of sensitive and specific approaches which have especially contributed to solve inconclusive cases, thus enabling implementation of early and adequate treatment. The establishment of a gold-standard immunological or molecular diagnosis is still a challenge, mainly because of a lack of standardization among laboratories and also structural/economic concerns. In fact, the complexity of leishmaniases also acts as an important barrier to be surpassed. Therefore, we propose a combination of different techniques for maximizing sensitivity and specificity, for a safe and reliable diagnosis: for CL, when MST is positive and parasitological analysis is negative (or is not performed), qPCR and/or FC could be employed as complementary investigation. For VL, when clinical evaluation is indicative (after differential diagnoses) and screening serological tests show negative results, qPCR could be employed as an alternative exam performed in reference centers, thus avoiding the painful and invasive bone marrow biopsy, especially for children. For immunosuppressed individuals such as HIV/VL co-infected patients, the monitoring of parasite load through qPCR is fundamental to predict relapses as well as treatment failures.

## Conclusions

Immunological tools are options in leishmaniases diagnosis, highlighting the growth of FC as a more specific and sensitive alternative to overcome the limitations of these techniques and even as a method to assess clinical cure. Likewise, molecular diagnosis has become a potential strategy to provide the early detection and consequently the fast treatment implementation, as well as for species characterization, assessment of treatment efficacy and monitoring of relapses. The easiness, safety and high accuracy have made the molecular biology increasingly interesting as complement or as alternative for defining the definitive VL and CL diagnosis. The technological advance in this case has becoming an adjuvant to prevent the continuous spread of the disease and its social consequences, mainly at poor populations.
